# Supplementary material for: Polyketide synthase gene clusters in the ericoid mycorrhizal fungus Oidiodendron maius: genome-wide prediction, expression profiling, and functional characterization of knockout mutants
Source: Front Plant Sci. 2026 Sep 16;17:1924430. doi: 10.3389/fpls.2026.1924430 (PMC13623903; doi:10.3389/fpls.2026.1924430)
Supplement: Supplementary file 1 [file SupplementaryFile1.zip › Supplementary Materials/Supplementary Folder 1.zip!/Oidma1_AssemblyScaffolds_Repeatmasked/knownclusterblast/cluster55/jgi.p_Oidma1_173692_mibig_hits.html]

| MIBiG Protein | Description | MIBiG Cluster | MiBiG Product | % ID | % Coverage | BLAST Score | E-value |
| --- | --- | --- | --- | --- | --- | --- | --- |
| EAL89349.1 | conserved\_hypothetical\_protein | BGC0001403 | Trypacidin\_biosynthetic\_gene\_cluster | 60 | 98.064516129 | 383 | 3.2e-106 |
| AGO59036.1 | PtaH | BGC0000121 | Pestheic\_acid\_biosynthetic\_gene\_cluster | 56 | 97.4193548387 | 366 | 3.1e-101 |
